# Risk of Vertical Transmission of Human Papillomavirus throughout Pregnancy: A Prospective Study

**DOI:** 10.1371/journal.pone.0066368

**Published:** 2013-06-13

**Authors:** Seung Mi Lee, Joong Shin Park, Errol R. Norwitz, Ja Nam Koo, Ig Hwan Oh, Jeong Woo Park, Sun Min Kim, Yun Hwan Kim, Chan-Wook Park, Yong Sang Song

**Affiliations:** 1 Department of Obstetrics and Gynecology, Seoul National University College of Medicine, Seoul, Korea; 2 Department of Obstetrics and Gynecology, Seoul Metropolitan Government Seoul National University Boramae Medical Center, Seoul, Korea; 3 Department of Obstetrics and Gynecology, Tufts University School of Medicine, Boston, Massachusetts, United States of America; 4 Seoul Women's Hospital, Incheon, Korea; 5 Department of Obstetrics and Gynecology, Inje University College of Medicine, Ilsan-Paik Hospital, Gyeonggi, Korea; 6 Department of Obstetrics and Gynecology, Ewha Womans University School of Medicine, Seoul, Korea; 7 Cancer Research Institute, Seoul National University College of Medicine, Seoul, Korea; 8 Major in Biomodulation, World Class University, Seoul National University, Seoul, Korea; Albert Einstein College of Medicine, United States of America

## Abstract

**Objective:**

Much controversy still exists about maternal-to-infant transmission of human papillomavirus (HPV) infection, specifically about the magnitude of the risk and the route and timing of such vertical transmission. This prospective cohort study examines the risk of vertical transmission of maternal HPV in each trimester of pregnancy.

**Study design:**

One hundred fifty three healthy pregnant women were followed longitudinally throughout pregnancy and cervical swabs obtained in each trimester and postpartum for HPV detection. Cord blood, neonatal nasopharyngeal aspirates, and placental biopsies were collected at delivery. DNA isolation, polymerase chain reaction, and hybridization were performed using the GG HPV Genotyping Chip Kit (Goodgene Inc., Seoul, Korea). Detection of HPV in neonates was defined as the presence of HPV DNA in either cord blood or neonatal nasopharyngeal aspirate.

**Results:**

HPV DNA was detected in 14%(22/153) of healthy women in the first trimester, 18%(22/124) in the second trimester, and 10%(15/153) in the third trimester; 24%(37/153) were positive for HPV DNA on at least one occasion in pregnancy. At birth, 5.2%(8/153) of neonates were HPV DNA positive. Seven of these eight infants were born to HPV-positive mothers. Placental HPV DNA was positive in 3.3%(5/152) of cases, and all five cases were from mothers with at least one HPV-positive test. Detection of HPV DNA in neonates was associated with detection of HPV in mothers during any of the three trimesters of pregnancy.

**Conclusion:**

HPV DNA was detected at birth in 5.2%(8/153) of neonates born to healthy women, and was associated with the detection of HPV in mothers during any of the three trimesters of pregnancy.

## Introduction

Human papillomavirus (HPV) is a member of the papillomavirus family of viruses that infects stratified epithelium of the skin and mucous membranes. While the majority of the nearly 200 known HPV types cause no symptoms, 30–40 HPV types are capable of infecting the anogenital region and causing disease. HPV infection appears to be the central etiologic agent for the development of most cases of cervical cancer [1] as well as malignancies of several other organs including the vulva, vagina, anus, and oropharynx [2]–[9]. It is believed that HPV infections are spread almost exclusively by sexual contact, but the epidemiologic data investigating the association between sexual behavior, oropharyngeal HPV infections and cancer (such as age at first sexual intercourse and number of lifetime oral sexual partners) are inconsistent. For example, a considerable number of such patients report only a few lifetime sexual partners and no history of oral sexual behavior [7]–[9]. These data suggest that other routes of transmission may be involved.

Vertical transmission of HPV from mother to fetus is known to occur. Indeed, up to 80% of neonates born to women with genital HPV have HPV DNA detectable in their nasopharyneal aspirate or oral mucosa [10]–[12], and this may persist for months or years. Cason et al reported that, among infants who were positive for HPV-16 at birth, HPV-16 DNA could still be detected in 60% of infants at 6 months of age [12]. And in other reports, HPV was noted to be persistent in the oral mucosa in 10% of infants at 3 years of age [13]. Despite a high prevalence of HPV DNA detection, clinical disease in children is uncommon. Perinatal transmission of HPV types 6 and 11 can lead to the development of juvenile-onset recurrent respiratory papillomatosis (JORRP) [14], but this is rare with an estimated annual incidence of two to four per 100,000 infants [15], [16].

There is still much controversy about maternal-to-fetal transmission of HPV, specifically about the magnitude of the risk and the route and timing of such vertical transmission. This prospective cohort study examines the risk of vertical transmission of maternal HPV in each trimester of pregnancy.

## Methods

### Study design

Healthy women with a singleton pregnancy presenting in the first trimester (<14 weeks of gestation) to a single primary obstetric care clinic (Incheon Seoul Women Hospital) in Incheon, Korea between May 2009 and June 2010 were approached for inclusion in the study. The decision of whether or not to participate was at the patient's discretion, and the patient was not disadvantaged in any other way for declining participation in the study. Subjects were excluded if they had evidence of immunosuppression (such as HIV infection, transplantation, or malignancy), a connective tissue disorder, or were receiving medications that may affect immune function. Women were invited to participate whether or not they had a history of genital HPV infection. The study was approved by the local institutional review board (SNUCM-SNUH IRB, Seoul National University College of Medicine-Seoul National University Hospital Institutional Review Board), and written informed consent was obtained from all study participants.

Each participant was asked to provide a comprehensive medical history and to complete a detailed demographic and medical questionnaire. Thereafter, all subjects underwent a routine obstetric examination. In all cases, cervical swabs were collected for HPV DNA testing (SurePath Technology; TriPath Imaging, Burlington, NC) at four time points in pregnancy: at enrollment in the first trimester (<14 weeks, which was also sent for cervical cytology), at a routine follow-up visit during the second trimester (14–28 weeks), in the third trimester (>28 weeks), and in the postpartum period. The 2001 Bethesda System terminology was used to report the results of the cervical cytology [17]. Women with abnormal Pap test results were referred for colposcopic examination and biopsy at the discretion of attending physician.

### Sampling for detection of HPV in neonates and placenta

Immediately after delivery, a sample of cord blood was set aside and a neonatal nasopharyngeal aspirate was collected with a bulb syringe using sterile technique so as to avoid possible contamination. Detection of HPV in neonates was defined as the presence of HPV DNA in either cord blood or neonatal nasopharyngeal aspirate. To evaluate placental infection, placental biopsies were routinely obtained from both the central and peripheral portions of the placenta again using sterile technique to avoid contamination. Detection of HPV in placenta was defined as the presence of HPV DNA in at least one placental biopsy.

### Detection of HPV DNA

HPV DNA was detected using the GG HPV Genotyping Chip Kit (Goodgene, Seoul, Korea), which identifies 40 HPV types (HPV 6, 11, 16, 18, 26, 30–35, 39, 40, 42–45, 51–56, 58, 59, 61, 62, 66–70, 72, 73, 81–84, 90, and 91). Genomic DNA extraction, amplification, labeling and hybridization as well as analysis were performed according to the instructions of the manufacturer. In short, extracting genomic DNA was performed with LaboPass™ Tissue mini prep. Kit (COSMO GENETECH Products, Seoul, Korea). The primers chosen were in the L1 gene: primer Ll and L3. PCR with the primer L1 and L3 amplified about 200-base pair DNA fragment of all genotype of HPV. A mixture of 10 µL of HPVDNA-amplified product and 10 µL beta-globin-amplified products were denatured by heating at 95°C for two min, followed by cooling for 3 min on ice. The samples were mixed with 65 µL of hybridization buffer (Goodgene, Seoul, Korea) and put on the HPV DNA chip. The HPV DNA chip was incubated at 50°C for 30 min. And then the HPV DNA chip was washed twice with 3× SSPE for 2 min and 1× SSPE for 2 min. This lead to the formation of visible spots on the array surface, which then were scanned, measured and analyzed using a dedicated reader and software after the chip was dried (Affymetrix 428 Array Scanner, Molecular Devices Inc., California, USA).

### Statistical methods

Proportions were compared using the Fisher's exact test, and comparisons of continuous variables between groups were performed with a Mann-Whitney U test. Multivariate logistic regression analysis was used to adjust for potential confounding variables. p<0.05 was considered statistically significant. The SPSS software package (SPSS, Chicago, IL) was used for all analyses.

## Results

### Study population

Of the 384 women originally enrolled in the study, 199 were followed throughout pregnancy. Of these, 46 subjects were excluded from the analysis (three because they delivered at another institution, and 43 because sampling of neonatal nasopharyngeal aspirate and/or cord blood were not done). The remaining 153 cases were included in the final analysis. Among these, cervical HPV testing was performed in all three trimesters in 124 cases, and only in first and third trimesters in 29 cases.

At birth, 5.2% (8/153) of neonates were HPV DNA positive. Among these eight neonates, six were HPV positive in nasopharyngeal aspirate and two in cord blood. Table 1 shows the characteristics of study population. The risk of HPV detection was not increased in neonates who were delivered vaginally; indeed, in this cohort, a significantly higher percentage of neonates who were positive for HPV DNA were delivered by cesarean as compared with those who were negative for HPV DNA (63% [5/8] vs 28% [40/145], respectively; p<0.05) (Table 1). The indications for cesarean delivery in the 5 women with HPV-positive neonates were: elective repeat cesarean (n = 1), failure to progress (n = 2), cephalopelvic disproportion (n = 1), and elective primary cesarean (n = 1).

**Table 1:** Characteristics of the study population.

		Neonatal status		
	Total population (n = 153)	HPV negative (n = 145)	HPV positive (n = 8)	p-value
Maternal age (years)†	31 (20–43)	31 (20–43)	29 (27–42)	0.23
Nulliparity	75 (49%)	69 (48%)	6 (75%)	0.16
Current or ex-smoker	26 (17%)	25 (17%)	1 (13%)	1.0
Alcohol use	31 (20%)	30 (20%)	1 (13%)	1.0
Gestational age at delivery (weeks)†	39.7 (35.3–41.4)	39.7 (35.3–41.4)	39.9 (37.6–41.3)	0.63
Birth weight (grams)†	3320 (1760–4920)	3300 (1760–4920)	3560 (3120–3660)	0.07
Neonatal sex (male)	77 (50%)	72 (50%)	5 (63%)	0.71
Cesarean delivery	45 (29%)	40 (28%)	5 (63%)	0.05

† values are given as median (range).

HPV: Human Papillomavirus.

### Detection of HPV in neonates and mothers

HPV DNA was detected in 14% (22/153) of healthy women in the first trimester, 18% (22/124) in the second trimester, and 10% (15/153) in the third trimester. HPV DNA was detected less frequently in third trimester than in first and second trimester (p<0.01 for each). A total of 24% (37/153) of women were positive for HPV DNA on at least one occasion in pregnancy.

All 153 women in the study population underwent cervical cytology (Pap smear) screening in the first trimester. Of these, 148 women had normal result, and 5 women had ASCUS. Among the 5 women with ASCUS, 2 were positive for high-risk HPV. One woman with high-risk HPV and ASCUS underwent colposcopy and cervical biopsy, which showed no evidence of cervical cancer. The other woman declined such testing, and a repeat Pap smear was normal.

Table 2 compares the frequency of HPV DNA detection in mothers and placentas according to the HPV status of the neonates. Detection of HPV DNA in neonates was associated with a significantly higher incidence of HPV-positive mothers in all three trimesters of pregnancy and with HPV-positive placentas. Overall, seven of eight HPV DNA-positive neonates had HPV DNA-positive mothers in antenatal period (Table 3), and this association remained significant after adjustment for confounding variables such as maternal age, smoking, alcohol use, and mode of delivery (OR, 28.7; 95% CI, 3.2–258.6; p<0.005, multivariate logistic regression analysis). Although the risk of detection of HPV DNA in neonates was associated also with HPV DNA-positive mothers in the postpartum period, this association did not reach statistical significance (Table 2). A linear association existed between the risk of HPV DNA detection in the neonates and the presence and duration of HPV DNA-positive period in the mothers: 1% (1/116) in women without detectable HPV DNA in pregnancy, 14% (3/21) in women with HPV DNA detected during only one trimester, and 25% (4/16) in women with HPV DNA detected during more than one trimester (p<0.001, chi-square test).

**Table 2:** Association between HPV status of the neonates and the detection of HPV in the mothers.

		Neonatal status			
	Total population (n = 153)	HPV negative (n = 145)	HPV positive (n = 8)	p-value	Adjusted p-value†
1^st^ trimester	22/153 (14%)	18/145 (12%)	4/8 (50%)	0.02	0.02
2^nd^ trimester	22/124 (18%)	18/118 (15%)	4/6 (67%)	0.01	0.00
3^rd^ trimester	15/153 (10%)	11/145 (8%)	4/8 (50%)	0.00	0.01
Antepartum maternal HPV Never	116/153 (76%)	115/145 (79%)	1/8 (13%)	0.00	0.01
1 trimester only	21/153 (14%)	18/145 (12%)	3/8 (38%)		
>1 trimester	16/153 (11%)	12/145 (8%)	4/8 (50%)		
Postpartum maternal HPV	10/106 (9%)	8/98 (8%)	2/8 (25%)	0.17	0.11
Placental HPV	5/152 (3%) *	2/144 (1%)	3/8 (38%)	0.00	0.00

* One placental sample was not sent for HPV DNA testing.

† Adjusted for maternal age, smoking, alcohol use, and mode of delivery.

HPV: Human Papillomavirus.

**Table 3:** Cases in which HPV DNA was detected in neonates.

Cases	Maternal age (years)	Neonatal HPV types		Placental HPV types	Gestational age at delivery (weeks)	Mode of delivery	Maternal HPV types			
		Nasopharyngeal aspirate	Cord blood				1^st^ trimester	2^nd^ trimester	3^rd^ trimester	Postpartum
1	27	84	(-)	(-)	39+6	CD	(-)	(-)	(-)	(-)
2	42	(-)	**59**	(-)	37+4	CD	**59**	(-)	(-)	(-)
3	29	**16**	(-)	(-)	40+6	VD	**16**, 6	**16**	**16**	(-)
4	31	83	(-)	(-)	39+2	VD	(-)	83	(-)	83
5	27	NA	**53**	**53**	39+6	VD	**53**	**53**	(-)	(-)
6	35	**56**	(-)	(-)	39+6	CD	(-)	**56**, 42	**56**	(-)
7	29	**56**	(-)	**56**	41+2	CD	(-)	NA	91	(-)
8	27	**39**	(-)	**39**	39+1	CD	**39**	NA	**39**	**39**

High-risk HPV are in bold.

NA: not available, CD: cesarean delivery, VD: vaginal delivery.

HPV: Human Papillomavirus.

**Figure 1:**
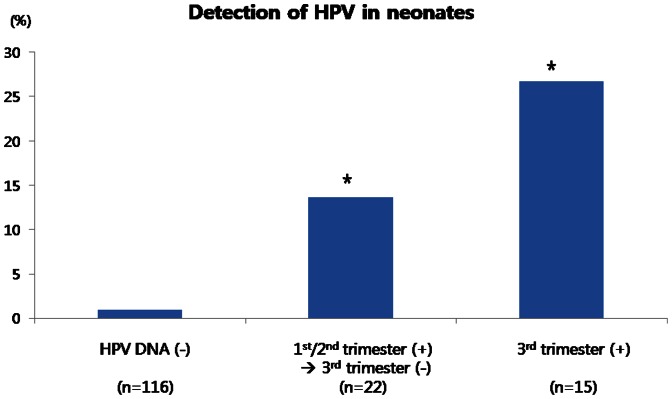
HPV DNA detection in neonates according to the presence or absence of HPV DNA in mothers during the antenatal period. HPV DNA (-): Mothers with no detectable HPV DNA at any point during pregnancy. 1^st^/2^nd^ trimester (+)→3^rd^ trimester (−): Mothers with detectable HPV DNA during either the 1^st^ or 2^nd^ trimester, which cleared in the 3^rd^ trimester. 3^rd^ trimester (+): Mothers with detectable HPV DNA during the 3^rd^ trimester. * p<0.05 compared with mothers without detectable HPV DNA during pregnancy. HPV: Human Papillomavirus.

Figure 1 shows the risk of HPV DNA detection in the neonates according to the HPV DNA status of the mothers during the antenatal period. Compared with neonates born to women without antenatal HPV DNA detection, the risk of HPV DNA detection in neonates was significantly higher in women who were HPV DNA positive during the first or second trimester which cleared in the third trimester, but were highest in neonates born to women who were HPV DNA positive during the third trimester (Figure 1). This increased risk remained significant after adjustment for confounding variables such as maternal age, smoking, alcohol use, and mode of delivery (OR, 35.4; 95% CI, 2.9–434.6; p<0.01, multivariate logistic regression analysis).

## Discussion

Much controversy still exists about maternal-to-fetal transmission of HPV, specifically about the magnitude of the risk and the route and timing of such vertical transmission. In this cohort of 153 normal pregnant women, HPV DNA was detected in 14% of women in first trimester, 18% in the second trimester, and 10% in the third trimester; 24% of women were positive for HPV DNA in at least one trimester of pregnancy. At birth, 5.2% (8/153) of neonates were positive for HPV DNA, and seven of these eight neonates had mothers who were positive for HPV DNA during pregnancy. Detection of HPV in neonates was associated not only with detection of HPV DNA in mother in the third trimester of pregnancy, but also in the first or second trimester.

Several studies have demonstrated an increased risk of HPV vertical transmission in neonates born to mothers at high risk, including women with a history of genital warts, abnormal Pap smears, or cervical dysplasia [10], [18], [19]. However, the risk of vertical transmission in low-risk pregnancies varies significantly between studies. Tenti et al reported a 30% risk of vertical transmission with a 1.5% risk of neonatal infection, all born to HPV-positive mothers [20]. Tseng et al reported similar results with a vertical transmission rate of 39.7% from HPV 16/18-positive mothers to their newborns [21]. In contrast, in two small studies, Smith et al [6] and Sarkola et al [22], found only a 7.7% (1/13) and 15.8% (3/19) risk of vertical transmission, respectively.

The strength of the current study is that, in contrast to all prior studies that looked only at HPV status in the third trimester and mother-to-fetal transmission, we followed low-risk pregnant women with HPV testing longitudinally throughout all three trimesters of pregnancy and in the postpartum period. In the current study, 88% (7/8) of newborns who were positive for HPV had mothers with antenatal HPV DNA detection. As shown in Table 3, only three out of eight neonates positive for HPV had the same HPV types as that detected in their mothers in the third trimester of pregnancy. However, when comparing neonatal and maternal HPV types across all gestational ages, the concordance rate of HPV type is 75% (6/8) (Table 3). This observation provides additional evidence in support of the hypothesis that vertical transmission may occur early in pregnancy.

Among 8 neonates who were HPV DNA positive, one neonate had different HPV type to its mother and another was born to mother without detectable antenatal HPV DNA. This discrepancy may be explained by contamination of the sampling or PCR technique (but these explanations are unlikely due to the methodology used to prevent contamination during sample collections and analysis), or by vertical transmission at other times during pregnancy when maternal sampling for HPV was not done. As we collected maternal samples for HPV DNA detection at only three time points during pregnancy, we may have missed the critical maternal HPV infection responsible for the vertical transmission, which was then cleared from the genital tract by the maternal immune system before the next HPV test.

The precise route of vertical transmission remains unclear. Several studies have reported that the risk of vertical transmission is increased with vaginal delivery, suggesting that perinatal transmission occurs as the fetus passes through an infected birth canal [6], [23], [24]. However, other investigators have reported that cesarean delivery is not protective against perinatal transmission [22], suggesting that route of delivery may not be a critical risk factor. In the current study, the risk of HPV DNA detection in neonates was not significantly reduced in women delivered by cesarean delivery. Indeed, more HPV DNA-positive neonates were born by cesarean than vaginal delivery (p<0.05, Table 1). These data suggest that other routes of vertical transmission may exist. These may include periconceptual transmission and/or antenatal transmission (through either transplacental hematogenous transmission or ascending infection) or through amniotic fluid [13], [25], [26]. Tseng et al favored transplacental hematogenous transmission, because they detected HPV 16 DNA in 14% of cord blood and showed that HPV DNA in cord blood was more closely related to the maternal HPV DNA status in peripheral blood mononuclear cells than that in cervicovaginal cells [27]. In contrast, Sarkola et al favored ascending intrauterine infection, because they detected HPV DNA in 4.2 and 3.5% of placenta and cord blood samples, respectively, and localized HPV DNA in placental syncytiotrophoblastics using in situ hybridization [22]. In addition, Armbruster-Moraes identified HPV DNA in amniotic fluid samples in the presence of intact fetal membranes in patients with abnormal Pap smears, suggesting that the virus may be able to pass across the fetal membranes and be transmitted to the fetus via the amniotic fluid [28].

In the current study, we measured HPV DNA in the maternal genital tract using a cervical swab. This should be distinguished from serostatus, which refers to evidence of active or past infection [29].

We considered neonates positive for HPV if cord blood or nasopharyngeal aspirate tested positive for HPV DNA. This definition may over-estimate the actual burden in neonates, since it does not identify live virus [30]. In addition, the HPV test is a qualitative and not a quantitative test; as such, we were not able to measure viral load in the study population. Further investigation is needed to determine whether or not HPV vertical transmission is related to the viral load in the maternal genital tract.

The clinical significance of HPV DNA detection in neonates also needs to be further investigated. The presence of HPV DNA in nasopharyngeal aspirate may represent infection or simply exposure. In the prior studies of Park et al. and Rombaldi et al., all infants who were HPV positive at birth subsequently tested HPV negative at both 6 months and 12 months of life [10], [31]. However, other reports have shown that HPV can be detected persistently in infants for months and years after birth, suggesting that this is not simply contamination with maternal virus [12], [13]. Although HPV is known to cause clinical disease, including benign orogenital lesions (warts) as well as cancer of the cervix and head and neck, it is still unclear how often infantile HPV infection progresses on to clinical disease [4]. If vertical transmission does occur and if the virus can persist in a dormant state for many years, then mother-to-fetal transmission may be laying the blueprint for cancer development later in life in many organ systems.

It has recently been suggested that a subset of head and neck squamous cancers, especially oropharyngeal cancer, is HPV-related [5]. Indeed, 40–80% of oropharyngeal squamous cell cancers in the US are positive for HPV 16 [2]–[5]. In spite of the importance of HPV in oropharyngeal cancer, the epidemiology and natural history of oral HPV infection is not well understood. Evidence suggests that the transmission of oral HPV is related to sexual behavior, because risk factors for HPV-positive head and neck cancer include early age at first sexual intercourse and a large number of lifetime oral sexual partners [7]–[9]. However, since 40% of oropharyngeal cancers have less than 3 lifetime sexual partners and 8–40% have no history of oral sexual behavior, other route of transmission should be also considered, [7], [8]. Vertical transmission from mother to fetus might be one such source of oral HPV infection. Up to 80% of neonates born to mothers with genital HPV are themselves positive for HPV in nasopharyneal aspirate or oral mucosa [10]–[12]. Until now, there has been a paucity of information regarding this relationship, and little is known about the clinical significance of HPV detection in neonates or how such infections can be prevented by HPV vaccination. Although only one neonate in the current study was positive for HPV-16 DNA (0.7% (1/153)), which is the most commonly found type in oropharyngeal cancer, this low prevalence may be due to the fact that we enrolled low-risk pregnant women, only 2 of whom were positive for HPV-16. The study by Cason et al enrolled pregnant women with a prevalence of HPV-16 infection as high as 69% and in this cohort, vertical transmission of HPV-16 was reported as 73% at 24 hours after birth, of which 83% persisted to 6 months of age [12].

In conclusion, HPV DNA can be detected in 5% of neonates born to healthy women, and is associated with detection of HPV in mothers during any of the three trimesters of pregnancy. This observation has major implications for our understanding of the route and timing of HPV mother-to-fetal transmission.
